# Paediatric autoimmune diseases with ELANE mutations associated with neutropenia

**DOI:** 10.1186/s12969-023-00824-9

**Published:** 2023-04-28

**Authors:** Dan Zhang, Gaixiu Su, Sheng Hao, Jianming Lai, Shunqiao Feng

**Affiliations:** 1 Department of Rheumatology and Immunology, Capital Institute of Pediatrics, 2 Yabao Road, Chaoyang District, Beijing, China; 2 Department of Nephrology, Rheumatology and Immunology, Children’s Hospital of Shanghai (also known as Children’s Hospital, Shanghai Jiao Tong University School of Medicine), Shanghai, China; 3 Department of Hematopathology, Capital Institute of Pediatrics, Beijing, China

**Keywords:** ELANE mutations, Autoimmune diseases, Paediatric neutropenia

## Abstract

**Objective:**

To explore the clinical characteristics of autoimmune diseases in children with ELANE mutations.

**Methods:**

Three cases of children with ELANE mutations manifesting as autoimmune diseases, who were under treatment from April 2020 to May 2021, were retrospectively analysed.

**Results:**

Among the three children, two were boys aged 15 years and 22 months (cases 1 and 3) respectively, and the other one was a 22-month-old girl (case 2). All the cases had recurrent infections. Case 1 presented with cyclic neutropenia and systemic lupus erythematosus (SLE). Case 2 presented with severe neutropenia and autoimmune haemolytic anaemia (AHIA). Case 3 presented with severe neutropenia and anti-neutrophil cytoplasm antibodies (ANCA)-associated small vasculitis. Genetic tests showed that they all had heterozygous mutations in the ELANE gene. Case 1 was treated with methylprednisolone and hydroxychloroquine sulphate for 2 years, making neutrophil level return to normal. Case 2 received allogeneic hematopoietic stem cell transplantation and has stopped taking antibiotics, steroids and all the immunosuppressors. Case 3 received subcutaneous injections of granulocyte colony-stimulating factor, oral prednisone and cyclophosphamide. The boy in case 3 has been followed up for one year, and his absolute neutrophil count has increased to 1.56 × 10^9^/L.

**Conclusion:**

Patients with ELANE mutations, combined with autoimmune diseases, may have recurrent infections. Disease-modifying antirheumatic drugs (DMARDs) are effective for autoimmune diseases. Autoimmune diseases with ELANE mutations associated with neutropenia can be cured through allogeneic hematopoietic stem cell transplantation.

## Impact

1. Neutropenia is considered an inherent immune deficiency, while systemic lupus erythematosus (SLE), autoimmune haemolytic anaemia(AIHA)and anti-neutrophil cytoplasmic autoantibody (ANCA)-associated small vasculitis are considered classical autoimmune diseases.
2. These two types of immune disorders can be manifested in the same disease, so neutrophils may affect T and B lymphocyte differentiation.
3. Different mutation sites of the elastase, neutrophil expressed (ELANE) gene have different effects on neutrophil elastase (NE), which may be the reason for different clinical phenotypes.

## Background

ELANE mutations can be associated with a variety of immunodeficiency diseases such as severe congenital neutropenia (SCN) and cyclic neutropenia (CN), which are two main clinical phenotypes related to neutropenia. Compared to SCN, CN seemed to be more common. SCN and CN could present with fever, mucosal ulceration, lymphadenitis and severe and/or recurrent infections. However, autoimmune symptoms associated with ELANE mutations are rare. In this study, we reported three cases of ELANE mutations with associated autoimmune diseases such as SLE, AIHA, and ANCA-associated small vasculitis respectively. All the cases presented with SCN or CN.

## Methods

We retrospectively analysed the clinical presentation and genetic data of three children with ELANE gene deficiency presented with autoimmune diseases. The three, two boys and one girl, were treated at Children’s Hospital Affiliated to Capital Institute of Pediatrics, and Children’s Hospital of Shanghai (also known as Children’s Hospital Affiliated to Shanghai Jiao Tong University School of Medicine) between April 2020 and May 2021. Their medical history was collected, including age of onset, time to diagnosis, past illnesses, physical examination, laboratory tests, and whole exome genetic tests.

Main outcome measures: (1) Clinical manifestations; (2) Laboratory tests; (3) Imaging findings; (4) Whole exome genetic tests. This study was approved by the Ethics Committee at the Capital Institute of Pediatrics, and the legal guardians of the three children signed the informed consent.

## Results

The clinical manifestations of the three children are summarised in Table 1.

**Table 1:** Clinical manifestation of 3 patient

	Age	Gender	Duration of disease	Fever	oral ulcer	Lymphadenitis	Rash	Etiology	Infection site	ANC periodicity	Combined with antoimmune disease	Renal disorder	Hematological disorder
Patient 1	15 years old	male	6 months	+	-	+	+	Tuberculosis、EBV	Septicopyemia, gastrointestinal infection	3 weeks	SLE	+	+
Patient 2	1 year and 10 months old	female	More than 1 years	+	-	-	-	Staphylococcus aureus	Pneumonia, Otitis media	none	AIH	-	+
Patient 3	1 year and 10 months old	male	1 months	+	-	-	-	Preziosi water bacteria, Bacteroides forsythus, Streptococcus anginosus、Streptococcus mitis, Klebsiella pneumoniae, Micromonas micros, EBV	Omphalitis、Pneumonia	13 to 17days	ANCA-associated small vasculitis	-	+

### Case 1

was a 15-year-old boy with no siblings. He was admitted to the hospital because of “recurrent fever and rash for more than 6 months”. Laboratory test results: white blood count 2.92 × 109/L, C-reactive protein 7 mg/L, erythrocyte sedimentation rate 55 mm/H, protein in urine ++, 24-hour urine protein 740 mg, antinuclear antibody (ANA) 1:160, anti-Sm antibody positive, anti-SSA antibody positive, anti-U1RNP antibody positive, lupus anticoagulant positive, anti-β2 glycoprotein I-IgG positive, complement C3 0.392 g/L, complement C4 0.0286 g/L, renal biopsy: class V lupus nephritis. Accordingly, he was diagnosed with systemic lupus erythematosus. Medication for him: intravenous methylprednisolone 40 mg q12h and oral hydroxychloroquine 0.2 g. His fever and rash were relieved and he was discharged on prednisone oral 60 mg once per day. After four months of prednisone reduction, by 5 mg per month, the dosage was reduced to 40 mg and his fever relapsed, accompanied by cervical lymph node enlargement. The lymph node biopsy was positive on acid-fast Mycobacterium fluorescence staining (9/HP). The patient was considered to have lymphatic tuberculosis, and became afebrile after taking isoniazid and rifampicin. However, initial resolution of fever was followed by fevers occurring once every 3 weeks. During these episodes, blood test showed white blood count was as low as 1.47 × 109/L, and neutrophil was as low as 0.75 × 109/L. The fever lasted around 5–7 days in each episode, and subsided after anti-inflammatory treatment. Whole exome sequencing showed that the boy had ELANE gene c.103 C > T (p.R35X) heterozygous mutation, and his father also had heterozygous mutation at the same locus. The boy had NF1 gene c.5425 C > T (p.R1809C) heterozygous mutation, and his mother also had heterozygous mutation at the same locus. Further questioning and feedback revealed that the father had recurrent respiratory infection, which responded to antibiotics, and neutropenia,, and he had cyclic neutropenia on dynamic monitoring with no treatment. Based on the medical history, family health history and genetic testing results, the patient in case 1 was diagnosed with recurrent neutropenia in addition to systemic lupus erythematosus. He was given oral methylprednisolone, combined with cyclosporine and hydroxychloroquine. Methylprednisolone gradually tapered to, 8 mg/d (reduce dose by 4 mg per month), and then to 4 mg/d (reduce dose by 2 mg per month). He was followed up for two years. In terms of blood tests after hospital discharge, he had neutropenia about every 21 days, which can be relieved by oral Leucogen. No recurrent fever or serious infection occurred.

**Fig. 1:**
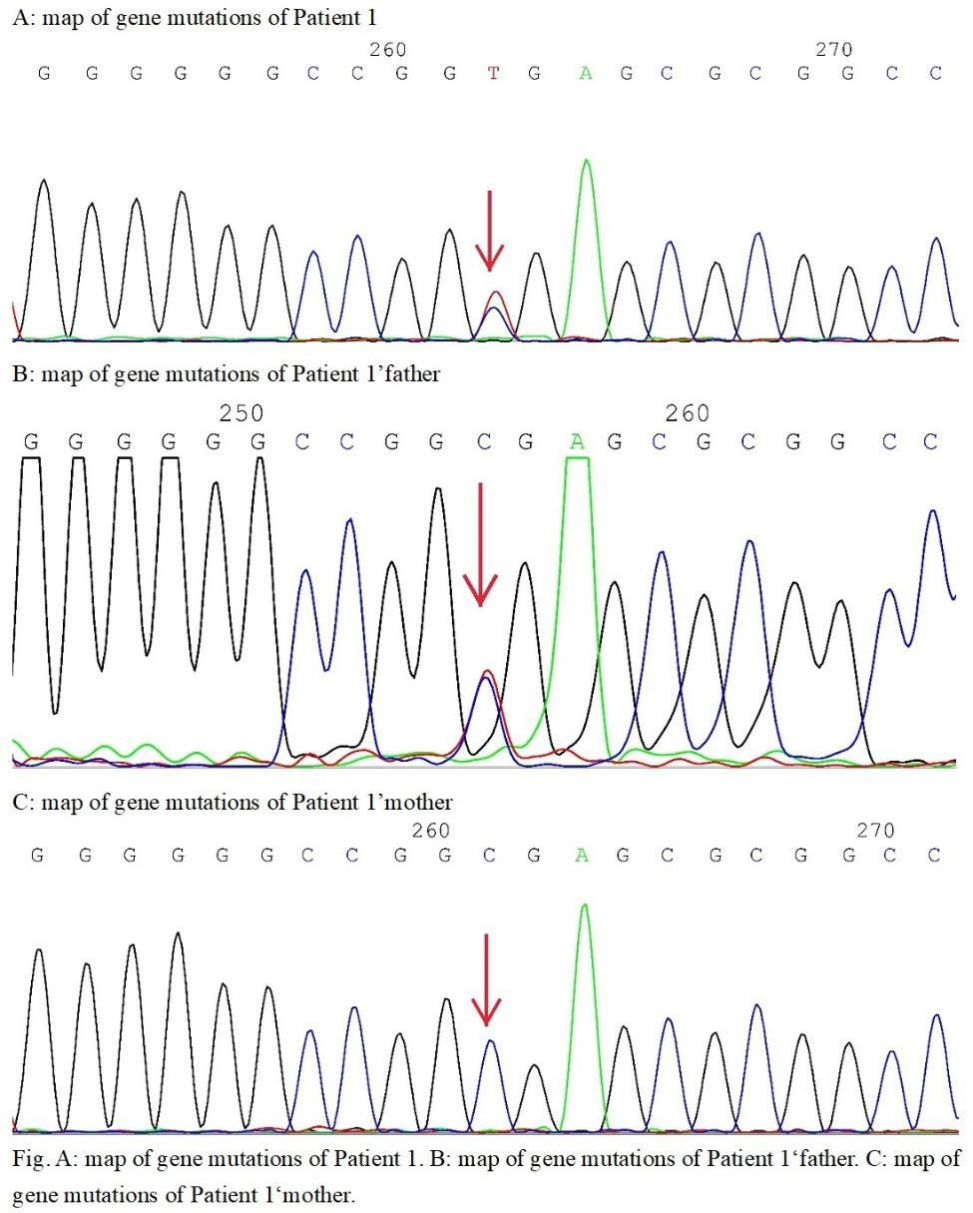
A: map of gene mutations of Patient 1. B: map of gene mutations of Patient 1’father. C: map of gene mutations of Patient 1’mother

### Case 2

was a 22-month-old girl with no siblings. She was admitted to the hospital mainly because of “repeated neutropenia for more than one year”. More than one year before admission, she came to a local hospital because of fever and purulent discharge of right ear. Her laboratory test results at the time: white blood count 6.1-10.55 × 10^9^/L, neutrophils 0.07–0.51 × 10^9^/L, haemoglobin 81-107 g/L, and platelet 584–770 × 10^9^/L. She was treated with antibiotics for otitis media and recovered. However, she continued to have recurrent infections including respiratory tract infection, otitis media, and lymphadenitis, which didn’t respond well to anti-infection therapy. Blood tests at the time showed neutropenia. She underwent bronchoalveolar lavage (BAL) due to presumed pneumonia and cultures were positive for staphylococcus aureus. Bone marrow tests revealed diminished granulocyte proliferation. Whole exome sequencing of bone marrow showed ELANE gene c.452G > T (p.C151F). Next-generation sequencing of oral mucosa indicated germline mutation. Physical examinations after admission showed positive perinuclear anti-neutrophil cytoplasmic antibodies (p-ANCA) positive, an elevated IgG level of 30.4 g/L, and Coombs test ++, indicating autoimmune haemolytic anaemia. Based on medical history and genetic tests, she was diagnosed with neutropenia and autoimmune haemolytic anaemia. The patient was treated with allogeneic hematopoietic stem cell transplantation. Thirty-eight days after transplantation, the patient developed rash and bloody stool, indicating graft versus host disease (GVHD). She was given intravenous cyclosporine A, tacrolimus, and methylprednisolone, and oral mycophenolate mofetil and ruxolitinib for anti-rejection. After hospital discharge, methylprednisolone was gradually reduced and stopped, and the patient was given oral mycophenolate mofetil and ruxolitinib for anti-rejection, and compound sulfamethoxazole, fluconazole, and ganciclovir for infection prevention. After 2 years of follow-up, the patient stopped all drugs, and no repeated infection occurred. Coombs test returned to negative, and neutrophils returned to normal.

**Fig. 2:**
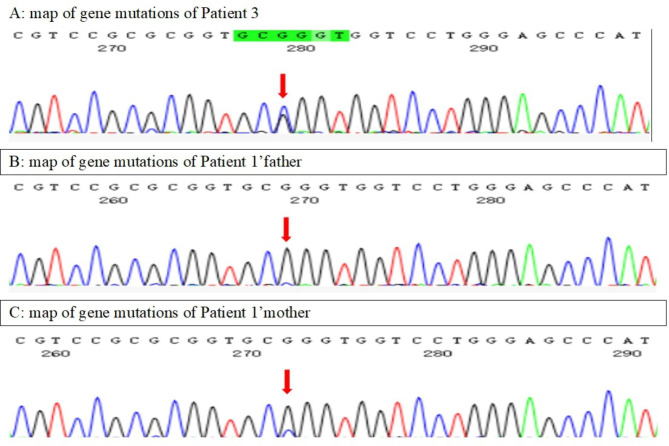
A: map of gene mutations of Patient 3. B: map of gene mutations of Patient 3’father. C: map of gene mutations of Patient 3’mother

### Case 3

was a 22-month-old boy with no siblings. He was admitted to the hospital here mainly because of “intermittent fever for 1 month and cough for 3 weeks”. Before admission here, blood tests at a local hospital showed neutropenia. Lung CT showed pulmonary lesions. He was given a variety of broad-spectrum antibiotics for infection, intravenous immune globulin, hydrocortisone, intermittent subcutaneous injection of granulocyte stimulating factor. However, he remained intermittently febrile despite these interventions. Medical history showed that three days after his birth, he was presented with fever and purulent discharge around umbilical cord, and was diagnosed with omphalitis that was cured by cephalosporin. He had suffered from Epstein-Barr virus (EBV) infection one year before admission, presented with fever, swollen lymph nodes and positive EBV DNA, which was cured by ganciclovir. Blood tests after admission showed white blood count 14.55 × 10^9^/L, neutrophil 0.04 × 10^9^/L, haemoglobin 102 g/L, platelet 442 × 10^9^/L, C-reactive protein 33 mg/L, and erythrocyte sedimentation rate 114 mm/H. He had the lowest neutrophil count about every 13–17 days. Urinalysis results were normal. He had an elevated IgG level of 20.1 g/L, complement C3 0.69 g/L, complement C4 0.12 g/L, ANA negative, Coombs negative, p-ANCA positive, cytoplasmic antineutrophil cytoplasmic antibody (c-ANCA) negative, myeloperoxidase antibodies (MPO) positive, proteinase 3 (PR3) negative, and anti-glomerular basal membrane antibody negative. Bone marrow cytology tests showed active bone marrow hyperplasia and markedly reduced granular hyperplasia. The next-generation sequencing (NGS) of bronchoalveolar lavage fluid tested positive for Prevotella, Bacteroides flexneri, Streptococcus angina, Streptococcus bradysiae, Klebsiella pneumoniae and Micromonas micros. Lung biopsy revealed hyperplasia fibrous connective tissue at some interstitial areas, and focal infiltration of numerous lymphocytes and plasma cells. Foam cell aggregation was seen in the alveolar lumen. Chest CT showed bilateral pulmonary infection, local abscess in the middle lobe of right lung, and bilateral pleura thickening and adhesion. The wall of the middle and lower oesophagus was thickened. Whole exome sequencing showed c.242G > C, p.R81P heterozygous mutation in the ELANE gene, which was inherited from the mother. Based on medical history and genetic testing results, he was diagnosed with cyclic neutropenia and ANCA associated vasculitis. He was given ceftriaxone, azithromycin for anti-infection coverage, 7.5 g immunoglobulin for three days, subcutaneous injection of granulocyte stimulating factor, prednisolone and cyclophosphamide. After treatment, his temperature returned to normal, along with a decrease of C-reactive protein and erythrocyte sedimentation rate. The patient was followed up for one year. His prednisone dose was tapered by 2.5 mg per month until 5 mg/d. Cyclophosphamide shock therapy was administered 7 times per month, and subcutaneous granulocyte colony-stimulating factor was given when the neutrophil count falls below 0.5 × 10^9^/L. The absolute value of neutrophils increased to 1.56 × 10^9^/L.

## Discussion

Consisting of five exons and four introns, the ELANE gene is found on chromosome 19p13.3. Its DNA sequence consists of about 5,000 base pairs. The ELANE gene encodes neutrophil elastase (NE), a chymotrypsin serine esterase synthesized during the transformation of myeloblasts to promyelocytic granulocytes. NE is part of the neutrophil serine proteases (NSP) complex (NSPs), which also includes PR3 and histone G [1–3]. NSPs is only expressed in mature myeloid monocytes and plays a key role in promoting inflammatory responses and the destruction of pathogens. Activation of the misfolded NE protein product and subsequent unfolded protein responses in accelerated apoptosis during myeloid cell differentiation, followed by a maturation arrest of neutrophil precursors at the promyelocytic/granulocyte stage.

Intracellularly, NE and lysosomes participate in microbial decomposition under the synergistic effect of antimicrobial peptide and nicotinamide adenine dinucleotide phosphate (NADPH) oxidase system [4]. Extracellularly, NE uses a variety of Gram-negative bacilli as substrates to split their pathogenic factors [5]. In inflammatory reactions, NE destroys bacteria and host tissues. Therefore, NE also plays an important role in non-infectious inflammatory responses [4]. Chromatin binds to positively charged NSPs or histones to form neutrophil extracellular traps (NETs) [6]. NETs capture pathogens through physical barriers, and NETs-related NSPs kill pathogenic microorganisms by degrading their virulence factors. Therefore, a lack of NE will weaken organism’s ability to clear pathogens.

Neutropenia is the main clinical manifestation of ELANE mutations. It has been reported that mutations in the ELANE gene are found in 80–100% of cyclic neutropenia (CN) cases [7–9] and 35–63% of severe neutropenia (SCN) cases [9–11]. Both spontaneous and germline mutations may occur. Neutropenia-associated ELANE mutations are usually frame shift and termination mutations that result in structural changes in protein.

CN is an autosomal dominant disease characterized by regular fluctuation of peripheral neutrophils from near normal to severe low levels, generally with a cycle of 21 days, but also can be 2–4 weeks [12]. Clinical manifestations include fever, lymphadenitis, oral ulcer and infections such as sinusitis, pharyngitis, cellulitis, pneumonia, acute peritonitis [10, 13, 14]. The pathogen of infection is usually Gram-negative bacilli. Symptoms usually recur, but severe infections, sepsis, and even death are rare. It can also be asymptomatic. SCN is a congenital phagocytic defect in primary immunodeficiency diseases, which can be autosomal dominant or recessive inheritance. Reported more than germline mutations, spontaneous mutations are characterized by repeated severe infections starting from first few months after birth. They may present with omphalitis at first, followed by otitis media, pneumonia, skin or liver abscess. Patients can have permanent teeth fall out at very early age due to stomatitis and gingival hyperplasia. Neutrophil absolute count (ANC) was always lower than 0.2 × 10^9^/L [10, 15]. The most common pathogens are Staphylococcus aureus and Gram-negative bacilli. Because SCN patients cannot produce pus, the absence of pus at infected lesions is a characteristic change of SCN. Bone marrow biopsy shows neutrophil precursor maturation stop at the promyelocytic/myeloid stage. Unlike CN, SCN is characterized by transition to myelodysplastic syndrome (MDS) or acute myeloid leukaemia (AML) [1, 16].

In our study, case 1 presented with recurrent fever, infection and paediatric systemic lupus erythematosus. His SLE was characterized by blood and kidney involvement, hypocomplementemia, and many autoantibodies tested positive, including high ANA, anti-Sm, anti-SSA, anti-U1RNP, lupus anticoagulant and anti-β2 glycoprotein I-IgG antibodies. Case 2 presented with repeated infection and autoimmune haemolytic anaemia. Case 3 presented with recurrent infections and ANCA-associated small vasculitis. The main manifestations of his ANCA-associated small vasculitis were pulmonary involvement and positive p-ANCA. All three patients had recurrent fever and infections. Case 1 had recurrent episodes, his fever and infections present at the same time, along with neutropenia. Infections in all three cases were mainly bacterial, along with Epstein-Barr virus infection in cases 1 and 3. Different from previous reports, none of the three had oral ulcers. According to literature, ELANE mutations can be associated with arthritis and pyoderma gangrenosum, but all of such mutations were reported as individual cases. There were no reports of SLE, ANCA-associated small vasculitis and autoimmune haemolytic anaemia. It has been reported that SCN caused by ELANE mutations can lead to myelodysplastic syndromes (MDS) or acute myeloid leukaemia (AML). However, in our study, cases 2 and 3 were presented as SCN, but no MDS or AML on bone marrow biopsy. All three cases in this group had germline mutations, and case 1 also had the neurofibromatosis type 1 (NF1) gene mutation. The ELANE mutation of case 1 came from his father, who also presented with CN, but no recurrent infections. The parents of cases 2 and 3 had no clinical presentation of CN or SCN.

Recombinant human granulocyte colony-stimulating factor (G-CSF) can elevate the ANC level to normal or nearly normal in CN patients. Low-dose G-CSF treatment can shorten the duration of neutropenia, but it cannot change the periodic nature of neutropenia. G-CSF is the first-choice treatment for SCN. The Severe Chronic Neutropenia International Registry (SCNIR) reported that more than 95% of patients respond to G-CSF treatment and ANC can increase to 1 × 10^9^/L. Most children with SCN respond to a dose between 3 and 10 µg/kg/day [17–19]. For patients who do not respond to G-CSF, hematopoietic stem cell transplantation is the only treatment at present [20]. The incidence of MDS or AML is 40% after 10 years in SCN patients treated with G-CSF greater than 8ug/kg/day. However, this complication is relatively rare in CN patients.

Although CN and SCN are mainly caused by ELANE mutations, studies reported that different sites of ELANE mutations can cause different clinical phenotypes. In other words, the clinical presentation of CN or SCN depends on the site of ELANE mutation and its effect on NE activity. The genotype and phenotype may have a one-to-one correlation. In this study, the three children had different genotypes, which led to different amino acid changes. Although two of them had SCN, they had different autoimmune diseases. The other case had CN, and the combined autoimmune disease was also different from the other two cases, which was consistent with the possible one-to-one correlation between genotype and phenotype reported previously.

Our literature review did not find clear pathogenesis of ELANE mutations combined with autoimmune diseases. However, it has been shown that dysregulation of innate immune pathways related to host defence has profound effects on various aspects of SLE pathogenesis, including disruption of immune tolerance, induction of interferon and other proinflammatory cytokines, abnormal adaptive immunity, and tissue damages. Evidence found in human-mouse models, both in vivo and in vitro, supports that neutrophil dysregulation plays a key role in the pathogenesis of SLE, including loss of immune tolerance, induction and amplification of inflammatory pathways, tissue damages, vascular diseases, and cardio metabolic dysfunction [21–24]. Studies reported that genetic diseases that change the cellular components of innate immunity have been shown to increase the risk and severity of SLE. ELANE mutations lead to neutropenia, which plays an important role in innate immunity, weakening anti-bacterial ability and effect in coagulation, angiogenesis, inflammation resolution and tissue repair. In addition, neutrophils can regulate innate and adaptive immune cells. The three children in this study were combined with SLE, AIHA and ANCA-related small vasculitis, respectively. These three diseases are all characterized by systemic damage caused by autoantibodies. Therefore, AIHA and ANCA-related small vasculitis may also have a mechanism similar to that of SLE.

Autoimmune symptom associated with ELANE mutations is rare. We may choose different prednisolone and immunosuppressive therapies for different diseases. Based on our study, we concluded that for CN patients, such as case 1, oral Leucogen and subcutaneous injection of G-CSF can improve neutropenia and reduce recurrent infections. For patients with CN and autoimmune diseases, we can give oral glucocorticoid and immunosuppressor, such as hydroxychloroquine and cyclosporine. For patients with autoimmune diseases and severe organ damages, intravenous cyclophosphamide may work. Allogeneic hematopoietic stem cell transplantation can cure immune deficiency and immune disorder completely for SCN patients such as cases 2 and 3, and is effective for patients with poor response to G-CSF or combined with MDS/AML.

## Conclusion

In conclusion, the ELANE mutation is a common cause of CN or SCN, with clinically manifestations ranging from recurrent fever, infections to oral ulcer, lymphadenitis. SCN may develop into MDS or AML, and autoimmune diseases are rarely reported. Subcutaneous injection of G-CSF and allogeneic hematopoietic stem cell transplantation can ameliorate neutropenia to a varying extent. If combined with autoimmune diseases, steroid and immunosuppressive therapy is effective. Special attention must be paid to children: When treating children with early onset problems, and recurrent fever and infections, clinicians should evaluate ANC and periodic patterns as this might indicate CN or SCN. If there are multi-system damages such as cytopenia, lung, kidney, and nervous system involvement, attention must be paid to the combination of autoimmune diseases. Whole exome sequencing should be considered for early diagnosis, so as to decide an appropriate treatment plan according to the child’s condition to ensure precise individualised treatment.

## Data Availability

The data that support the findings of this study are available from the corresponding author, Gaixiu Su, upon reasonable request.

## Abbreviations

SCN: severe congenital neutropenia
CN: cyclic neutropenia
SLE: systemic lupus erythematosus
GVHD: graft versus host disease
ELANE: ELAstase-Neutrophil Expressed
NE: neutrophil elastase
NSPs: NSP complex
PR3: proteinase 3
ANC: Neutrophil absolute value
MDS: myelodysplastic syndrome
AML: acute myeloid leukemia
G-CSF: granulocyte colony-stimulating factor
